# P-542. Long-Acting (LA) Intramuscular (IM) Cabotegravir and Rilpivirine (CAB/RPV) in Adults for the Maintenance of HIV-1 Suppression in County-Based Clinics in Riverside County

**DOI:** 10.1093/ofid/ofae631.741

**Published:** 2025-01-29

**Authors:** Chiao An Chiu, Marilyn La, Nikki Mulligan, Patrick Wu, Bruce Weng, Maily Hong, Bishoy Zakhary

**Affiliations:** Riverside University Health System Medical Center, Moreno Valley, California; Kaiser Permanente, Downey, California; Riverside University Health System Medical Center, Moreno Valley, California; Kaiser Permanente, Downey, California; Riverside University Health System Medical Center, Moreno Valley, California; Thomas J. Long School of Pharmacy, Stockton, California; Riverside University Health System Medical Center - Comparative Effectiveness & Clinical Outcomes Research Center, Moreno Valley, California

## Abstract

**Background:**

IM CAB/RPV effectively maintained HIV viral suppression in prior randomized controlled trials. The study aimed to apply CAB/RPV trial results in a county population. The secondary aim was to characterize socioeconomic factors affecting CAB/RPV implementation.

**Figure 1.**
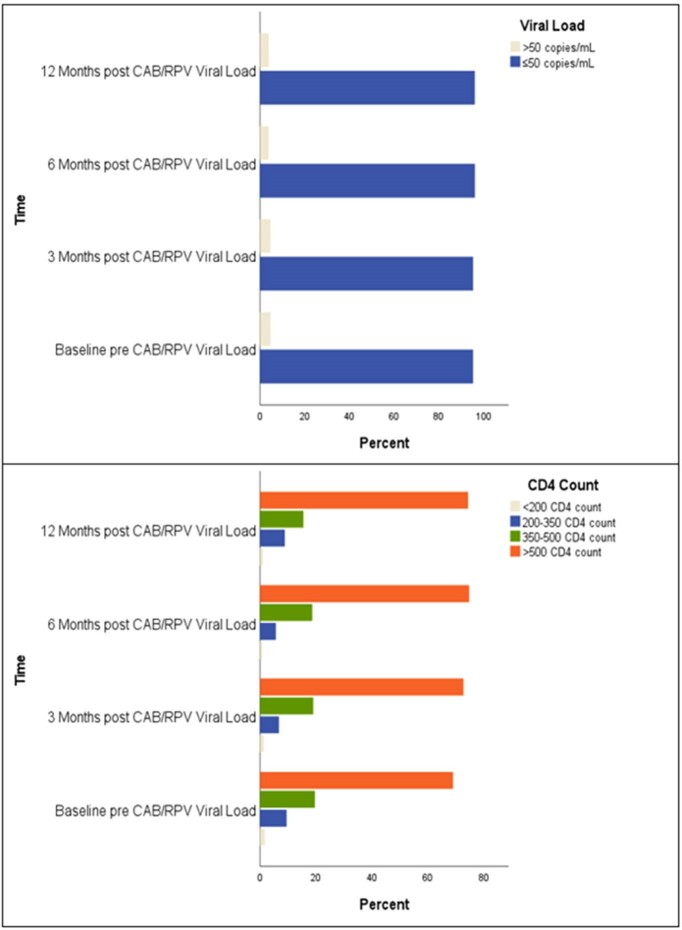
Baseline vs. 6-Month Post CAB/RPV Viral Load and CD4 (Top) HIV viral load between baseline, 3-month, 6-month, and 12-month post-CAB/RPV switch. (Bottom) CD4 count between baseline, 3-month, 6-month, and 12-month post-CAB/RPV switch

**Methods:**

We conducted a retrospective cohort study including adult HIV patients virologically suppressed on oral antiretroviral and switched to CAB/RPV. Patients received at least one oral or IM CAB/RPV dose from Riverside University Health System between January 2021 to 2024. We collected data by chart review. The primary outcome was the patient percentage of HIV-1 RNA ≤ 50 copies/mL. The secondary outcomes were virologic failure, CD4 count, adverse events, and adherence. We used McNemar’s test for categorical variables and Wilcoxon Signed-Rank test or paired t-test for continuous variables to compare outcomes at baseline and 6-month post CAB/RPV switch. We used univariant analysis to evaluate risk factors for the primary outcome. We used a binary logistic regression model to evaluate risk factors for discontinuation.

**Table 1.**
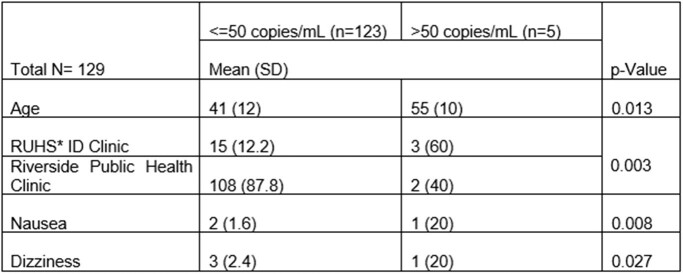
Risk Factors to HIV RNA level > 50 copies/mL at 6 months *RUHS= Riverside University Health System

**Results:**

We included 169 patients. 123/128 patients (96.1%) maintained HIV-1 RNA level ≤ 50 copies/mL at 6 months after CAB/RPV switch (p=0.727). CD4 count did not significantly change (p=0.115). Age, clinic type, and side effects were associated with HIV-1 RNA level > 50 copies/mL at 6 months. Virologic failure occurred in 5/128 patients (3.9%). 62/169 patients (36.7%) discontinued therapy. After controlling for demographic and clinical factors, a higher California Healthy Place Index (CA HPI), and longer distance to clinic were significant risk factors for discontinuation. There were more adverse events reported post-switch (52.7%, p< 0.001), the highest being injection site reactions (54/169, 32%). The number of rescheduled appointments was not different post-switch (70.3% vs 67.2%, p=0.683).

**Table 2.**
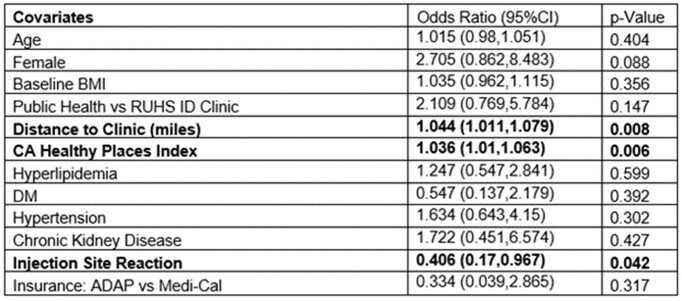
Binomial Logistic Regression Analysis for Therapy Discontinuation (n=197)

**Conclusion:**

96.1% of county patients maintained HIV viral suppression after CAB/RPV switch, consistent with prior trials. We observed a higher discontinuation rate compared to prior literature, associated with multiple socioeconomic factors. Resources for social and financial barriers are vital for CAB/RPV implementation to ensure treatment success and patient retention.

**Table 3.**
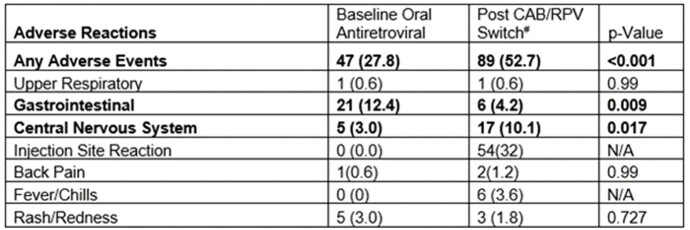
Baseline vs. 6-Month Post CAB/RPV Reported Adverse Reactions #Post CAB/RPV switch adverse events included all reported adverse events in patients who received at least one dose of oral or IM CAB/RPV

**Disclosures:**

**All Authors**: No reported disclosures

